# Beyond Thermal Conductivity: A Review of Nanofluids for Enhanced Energy Storage and Heat Transfer

**DOI:** 10.3390/nano15040302

**Published:** 2025-02-16

**Authors:** Ali Mirahmad, Ravi Shankar Kumar, Breogán Pato Doldán, Cristina Prieto Rios, Javier Díez-Sierra

**Affiliations:** 1 Iberian Center for Research in Energy Storage, Thermal Energy Storage Department, CIIAE, Avda. de las Letras, s/n, Campus University of Extremadura, 10003 Cáceres, Spain; ali.mirahmad@ciiae.org (A.M.); ravi.shankar@ciiae.org (R.S.K.); breogan.pato@ciiae.org (B.P.D.); 2 Department of Energy Engineering, University of Seville, Camino de los Descubrimientos, s/n, 41092 Seville, Spain; cprieto@us.es

**Keywords:** heat transfer fluid, nanofluid, nanoparticle, specific heat capacity, thermal conductivity, thermal energy storage, thermal management

## Abstract

The development of nanofluids (NFs) has significantly advanced the thermal performance of heat transfer fluids (HTFs) in heating and cooling applications. This review examines the synergistic effects of different nanoparticles (NPs)—including metallic, metallic oxide, and carbonaceous types—on the thermal conductivity (TC) and specific heat capacity (SHC) of base fluids like molecular, molten salts and ionic liquids. While adding NPs typically enhances TC and heat transfer, it can reduce SHC, posing challenges for energy storage and sustainable thermal management. Key factors such as NP composition, shape, size, concentration, and base fluid selection are analyzed to understand the mechanisms driving these improvements. The review also emphasizes the importance of interfacial interactions and proper NP dispersion for fluid stability. Strategies like optimizing NP formulations and utilizing solid–solid phase transitions are proposed to enhance both TC and SHC without significantly increasing viscosity, a common drawback in NFs. By balancing these properties, NFs hold great potential for renewable energy systems, particularly in improving energy storage efficiency. The review also outlines future research directions to overcome current challenges and expand the application of NFs in sustainable energy solutions, contributing to reduced carbon emissions.

## 1. Introduction

Over the past few decades, the demand for more efficient heat transfer processes has grown significantly, driven by technological advancements and the critical role of heat transfer fluids (HTFs) in modern energy production plants. Unfortunately, existing HTFs are unable to meet these increasing demands due to their limited thermophysical properties, particularly their low specific heat capacity (SHC) and thermal conductivity (TC). Common HTFs, such as molten salts and mineral oils, play essential roles in applications like solar thermal plants, but their SHC of less than 2 kJ/kg°C and TC of less than 1 W/mK hinder the overall efficiency of energy production systems. Nanofluids (NFs) present a promising opportunity to control and design various thermophysical properties, which can significantly enhance overall system performance. NFs are colloidal suspensions of NPs (1–100 nm) that exhibit exceptional characteristics, such as enhanced thermal properties compared to conventional fluids [1,2]. Raza et al. [3] investigated the impact of Al_2_O_3_ NPs and carbon nanotubes (CNTs) on the thermal performance of a direct absorption parabolic collector, observing efficiency improvements of up to 16% and 18%, respectively. Yaw et al. [4] examined a hybrid NF comprising graphene nanoplatelets and cellulose nanocrystals suspended in a 40:60 mixture of distilled water and ethylene glycol (EG) for engine cooling in automobiles. Their findings showed a 52% improvement in the convective heat transfer coefficient and a reduction in heat exchanger size, enabling lighter vehicle designs. Shafiei et al. [5] computationally optimized the cooling configuration of an electronic chip using SiO_2_-based NFs but found that increasing the NP concentration only slightly improved the cooling performance. Venkatesh et al. [6] investigated three different hybrid NFs—defined as the simultaneous use of different NPs in a single base fluid—including Al_2_O_3_/SiO_2_, SiO_2_/CuO, and Al_2_O_3_/CuO, for their impact on solar desalination with a flat plate collector. Among these, Al_2_O_3_/CuO stood out, making a modest 3% increase in TC but significantly boosting sweet water production by 41.7%.

**Figure 1:**
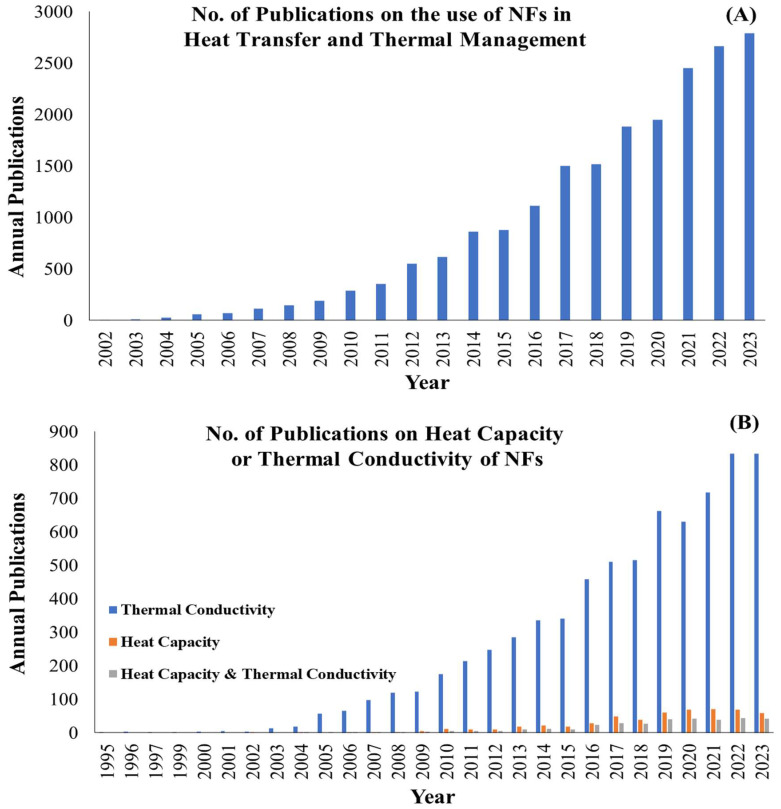
(**A**) Annual publications on the use of NFs for heat transfer and thermal management. (**B**) Annual publications on NFs with data on TC, SHC, and both TC and SHC simultaneously. Data sourced from Scopus.

Based on statistics reported by Scopus, Figure 1A depicts the growing attention to the use of NFs in heat transfer and thermal energy management applications. However, research on improving fluid properties often underlines either TC or SHC, with few studies considering both simultaneously, as is shown in Figure 1B.

**Figure 2:**
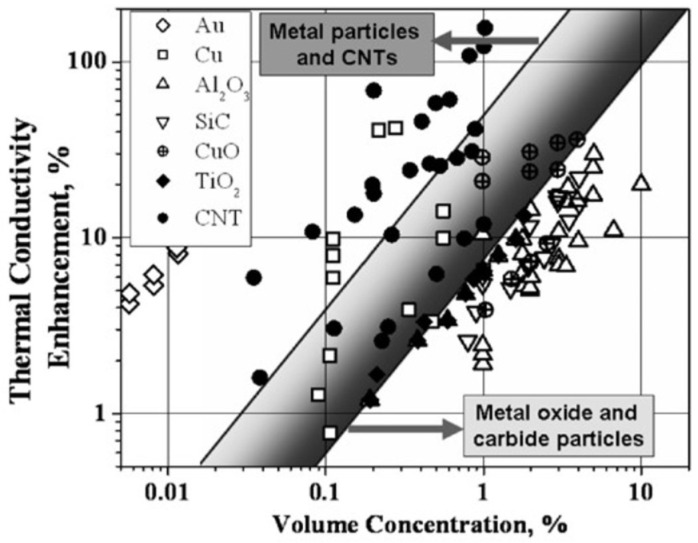
Comparative overview of TC enhancement with NP volume concentrations for various NP types [Reproduced with permission from ref. [46]. Copyright 2024, *KONA Powder and Particle Journal*].

A wide range of nanomaterials have been utilized to formulate NFs, including metals [1,7,8], carbon-based materials (single/multiwall nanotubes [9], graphenes [10]), and metal oxides [11,12,13,14]. These NPs have been produced in diverse shapes, such as spherical [15], cylindrical [15], rod-shaped [16], nanowires [17], nanotubes [18], nanoplatelets [10], and nanoplates [19]. Among the broad spectrum of NPs, metal oxides have been widely investigated for use in NFs despite usually having lower TC than pure metals due to their higher chemical stability (oxidation resistance), lower density, reduced toxicity, and ease of preparation [20]. Table 1 reports the thermophysical properties of a number of frequent NPs, and Figure 2 presents a comparative overview of several NF formulations with enhanced TC relative to the base fluid. The figure indicates that materials with high TC outperform those with lower TC.

The NPs have been dispersed in a variety of base fluids, including water [1], ethylene glycol (EG) [21], mineral oil [22,23], molten salts [24], ionic liquids [25], propanol [26], dimethylformamide (DMF) [27], silicone-based HTFs [28], perfluorohexane [29], and HCFC-141b [30]. These NFs have been explored for different operating temperatures, as summarized in Table 2.

**Table 1:** Thermophysical properties of nanoparticles.

Materials	Density (g/cm^3^)	Specific Heat Capacity (J/gK)	Thermal Conductivity (W/mK)
SiO_2_	2.4 [31]	0.745 [32]	1.4 [31]
CuO	6.5 [32]	0.536 [32]	33 [33]
TiO_2_	4.2 [32]	0.683 [32]	5.6 [33]
Al_2_O_3_	3.6 [34]	0.765–0.89 [34,35]	6.9–40 [34,36,37]
CNT	2.1 [34]	9.124 [34]	3007.4 [34]
Cu	8.933 [33]	-	400 [33]
MgO	3.58 [38]	0.877 [38]	48 [38]
ZnO	5.606 [38]	0.514 [38]	29 [38]
Ag	10.49 [31]	-	420 [31]
CaCO_3_	2.71 [31]	-	25.81 [31]

**Table 2:** Summary of the existing literature on NFs, including metal oxide NPs.

Base Fluid	NP	Shape	Size	Stabilizing Agent	Studied Temperature	Main Findings	Ref.
Water	TiO_2_	Spherical (Anatase)	15 nm	SDS ^1^	−24–−12 °C	For ice formation, rod-shaped TiO_2_ NPs have better performance because of higher crystallization temperature and enthalpy.	[39]
		Rod (Rutile)	Ø20 nm-L = 50 nm				
Water	Al_2_O_3_	Spherical	<20 nm	Chitosan	−20–0 °C	Adding NPs enhances water’s TC, while chitosan reduces the TC of the NF. The Al_2_O_3_ NPs play the role of promoting heterogeneous nucleation, temperature gradient elimination, and energy transmission during the phase change process of the NF.	[40]
BaCl_2_	TiO_2_	Spherical	20 nm	Hydrophilic Dispersant	−10–0 °C	The addition of TiO_2_ NPs accelerated the crystallization of the BaCl_2_ solution.	[41]
Water	TiO_2_	Spherical	95 nm	PVP ^2^	30–50 °C	PVP outperformed Tween 20 in stabilizing TiO_2_ NPs.	[42]
Water	TiO_2_	Spherical	20 nm	Chemical Surface Modification by GPTMS ^3^	25–60 °C	NF containing chemically modified TiO_2_ NPs showed higher TC compared with the use of commercial stabilizers like CTAB ^4^ and SDS.	[43]
Therminol VP-1	SiO_2_	Spherical	15 nm	No dispersant	100–220 °C	1 wt.% SiO_2_ increased the SHC of therminol oil by 5.41%.	[24]
Molten Hitec salt ^5^	Al_2_O_3_	Spherical	<50 nm	-	220–350 °C	The use of alumina NPs affects SHC non-uniformly, initially increasing it before decreasing below that of pure molten salt. At levelized temperatures, the NP effect weakens. The highest increase, 19.9%, occurs with 0.0625% NPs at 200–275 °C.	[44]
Eutectic of alkali chloride salts	SiO_2_	Spherical	27 nm	-	495–555 °C	The SHC elevation in the NF was attributed to the higher SHC of NPs compared to their bulk material, increased thermal resistance due to the larger NP surface area, and the formation of a layer at the solid–liquid interface.	[45]
Metal salt eutectic	SiO_2_	Spherical	15 nm	-	150–560 °C	2.5 wt.% SiO_2_ increased the SHC of metal salt by 14.59%.	[24]

^1^ Sodium dodecyl sulphate; ^2^ polyvinylpyrrolidone; ^3^ γ-glycidoxypropyltrimethoxysilane; ^4^ cetyl trimethyl ammonium bromide; and ^5^ including 53% KNO_3_ + 40% NaNO_2_ + 7% NaNO_3._

Generally, the synthesis of NFs can be performed using two methods: one-step and two-step. In the one-step method, NPs are formed and dispersed in the base fluid simultaneously, while in the two-step method, the NPs are first prepared separately and then dispersed into the base fluid [47,48] using physical methods such as sonication [49], agitation [50], and high shear homogenization [51]. The two-step approach is commonly utilized in NF synthesis due to its simplicity and controllability. In most cases, commercially available NPs are dispersed in a base fluid to form the NFs [10,37], or concentrated pre-prepared NFs are diluted to obtain the desired concentration [52].

**Figure 3:**
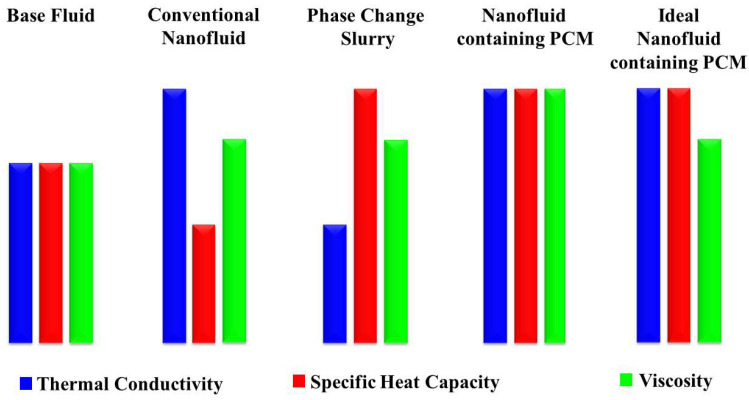
Qualitative comparison between various types of HTFs.

The thermal properties and performance of these NFs have been extensively studied in various applications. However, as shown in Figure 3, in most cases and despite improvement in TC, a drop in SHC is also observed in NFs [53,54]. On the other hand, several literature findings suggest the use of NPs for SHC enhancement, mainly using solid–liquid Phase Change Materials (PCMs) in phase change slurries. PCMs are substances that can isothermally absorb or release a high amount of heat by undergoing a transition from one phase to another [55,56,57]. However, their poor TC can diminish the TC of the formulated HTF [58]. As can be seen, research in NFs typically fails to balance SHC and TC, with limited studies aiming to enhance both [23]. This prompted the use of PCMs in NFs. However, the resulting NFs often experience a significant increase in viscosity. Ho et al. [59] prepared a water-based suspension of Al_2_O_3_ NPs and microencapsulated n-eicosane. Although they improved both SHC and TC, the viscosity of the formulated NF increased sharply by 200% [59]. This review articulates how NPs affect SHC and TC, both separately and together. Given the immaturity of the use of PCMs in NFs in improving both properties simultaneously, we focus on single-type NPs that can enhance SHC and TC while inhibiting pressure drop increases. Understanding these challenges and solutions could lead to better thermal management systems using new materials, helping to reduce energy gaps and carbon footprints during energy transitions.

## 2. Enhancing Thermal Conductivity of Nanofluids

**Figure 4:**
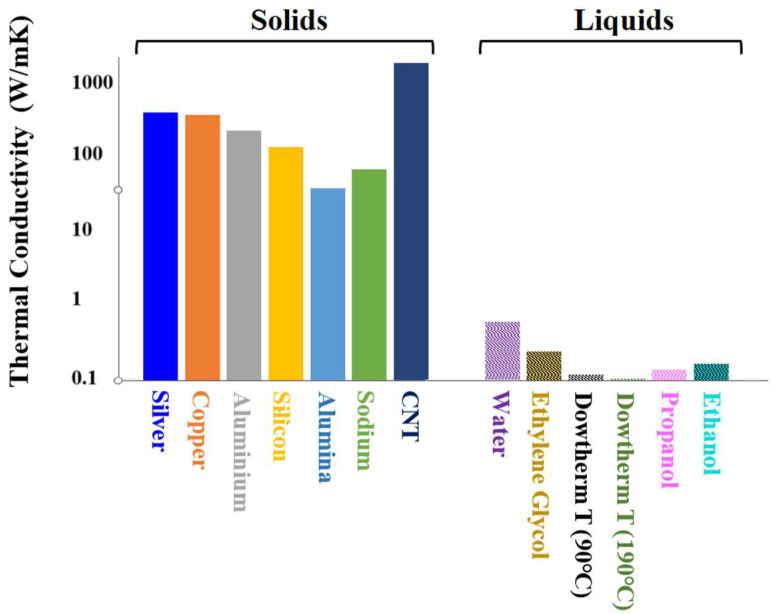
Comparison of TC values for various materials, showing how solid materials exhibit TCs that are several orders of magnitude higher than conventional fluids (in logarithmic scale).

**Figure 5:**
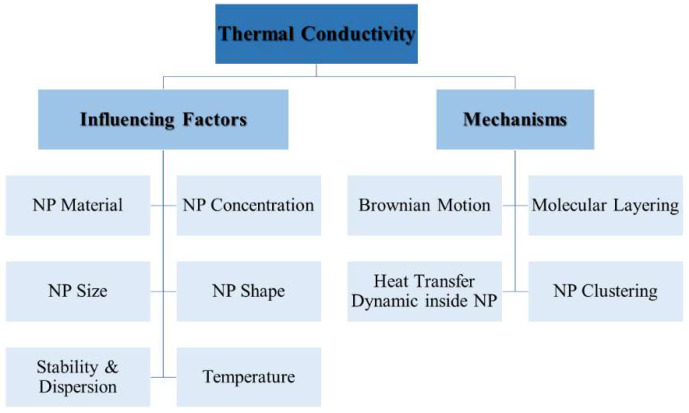
Mechanisms involved in TC enhancement of NFs and the influencing factors.

TC is a fundamental material property that quantifies the rate at which heat is conducted through a substance in response to a temperature gradient. It serves as a macroscopic indicator of the underlying molecular mechanisms that govern heat transfer within the material [60]. A higher TC value signifies an enhanced ability of the material to conduct heat, reflecting various factors such as molecular structure, bonding, and the arrangement of atoms or molecules. According to the data illustrated in Figure 4, even the TC of a solid electrical insulator such as Al_2_O_3_ is around two orders of magnitude larger than that of water [61]; it is expected that uniform suspension of metallic or metallic oxide particles in a base fluid will enhance its TC. However, the large discrepancies in the reported improvements in TC of NFs with respect to their base fluids as well as in the associated mechanisms indicate the complexity of the heat transfer phenomena in NFs [11,47,62]. The mechanisms involved and the parameters influencing TC enhancements are summarized in Figure 5.

### 2.1. Review on Thermal Conductivity of Nanofluids

Research on the TC of NFs under various conditions has yielded conflicting results. While some studies report enhancements that fall within the predictions of Effective Medium Theory (EMT), the majority exceed these predictions.

#### 2.1.1. Metallic NPs

Metallic NPs have been extensively studied as additives to enhance the TC of various base fluids, as metals are known for their high TC. However, these NPs often lack stability and may not be suitable for practical applications. Eastman et al. [21] synthesized an NF containing Cu NPs (0.3 vol%) suspended in EG, which improved the TC of the base fluid by 40%. Jana et al. [62] found that while Au NPs and Cu NPs individually performed well in water, their hybrid NF unexpectedly did not exhibit any synergistic enhancement of thermal conductivity. Xuan and Li [63] used 100 nm Cu NPs in water and transformer oil. They found that an 8 vol.% concentration of Cu NPs enhanced the TC of water by 70% and transformer oil by 40% [63]. Li et al. [64] stabilized Ag NPs in water using cationic Gemini surfactants and observed a non-linear increase in TC with rising suspension temperature and NP mass content. Nine et al. [65] investigated the stability of Cu and Ag NPs suspended in water. Initially, 1 mass% of Cu and Ag NPs increased TC by ~14% and ~12%, respectively. However, after the experiment, these improvements dropped to 4% for Cu and 10% for Ag, indicating greater instability in Cu NPs. Khamliche et al. [66] synthesized a copper–ethylene glycol NF using a single-step laser ablation method. Their best result showed a 24% improvement in TC of the base fluid, achieved with the sample subjected to a longer laser ablation time.

#### 2.1.2. Metallic Oxide NPs

Despite their lower TC compared to metallic NPs, metal oxide NPs have attracted attention for their potential to enhance the TC of NFs, primarily due to their lower density and superior stability [33]. Kong and Lee [67] evaluated the use of Al_2_O_3_–water NF in a double-pipe heat exchanger. They found that the addition of 1.3 vol.% Al_2_O_3_ NPs increased TC by 12%. Lee et al. [61] synthesized Al_2_O_3_ and CuO NPs using the gas condensation method and incorporated them into water and EG. Their experimental results proved that even small fractions of NPs can dramatically enhance the TC of the base fluid [61]. In the copper oxide/EG system, only 4 vol.% of 35 nm NPs increased the TC by more than 20% [61]. In another study, chemically precipitated Fe_3_O_4_ NPs were dispersed in water, showing a maximum TC enhancement of 48% with 2% Fe_3_O_4_ NPs but with a nearly threefold increase in viscosity as a drawback [68]. For the same Fe_3_O_4_–water NF system containing 0.024 vol.% NPs, Barai et al. [69] achieved an approximate 24% increase in TC.

Long et al. [41] used 1.13% TiO_2_ NPs in BaCl_2_ to improve low-temperature cool storage by storing 47.3% more cooling energy at the same time. He et al. [54] studied the similar NF for cold storage and shortened the freezing process by 64.5%. The rapid freezing was attributed to the enhanced TC of the solution, as it can further help in dissipating the heat released during crystallization [54]. Zhang et al. [43] achieved a nearly 20% improvement in TC by chemically modifying TiO_2_ NPs with γ-glycidoxypropyl trimethoxysilane and formulating a stable NF containing 2 vol.% NPs.

Vidal et al. [70] synthesized two grades of SiO_2_ NPs—one porous and one compact—and stabilized them separately in water as the base fluid. The porous NPs improved TC by 14.7%, while the compact NPs achieved only a 7.1% enhancement, demonstrating the superior performance of the porous NPs. Considering that CaCO_3_ NPs hydrolyze in water to yield Ca(OH)_2_, Selvan et al. [31] used CaCO_3_ NPs to stabilize SiO_2_ NPs in water. This approach resulted in a stabilized SiO_2_–water NF with a 31.84% improvement in TC.

#### 2.1.3. Carbonaceous NPs

Carbonaceous NPs combine the advantages of both metallic and metal oxide NPs. They have shown promising performance in NFs due to their low density, high stability, and positive impact on TC. In 2002, Choi et al. [47] managed to improve the TC of the oil by 150% using 1 vol.% of 25 nm CNTs and surpassed the values predicted using the Hamilton and Crosser (H&C) model [47]. Another study conducted by Xie et al. [15] also suggested the use of spherical and cylindrical silicon carbide (SiC) NPs in water and EG. By inserting 4.2% of spherical NPs and 4% of cylindrical NPs in base fluids, 15.8 and 22.9% improvements in TC were made, respectively. Opposing Lee et al. [61], the unexpected aspect of this research was the similar effect of NPs on both water and EG [15]. Despite their hydrophobic nature, untreated CNTs severely agglomerate in commercial oil, as observed by Marquis et al. [71]. They used succinimide as a dispersant to create stable suspensions of functionalized single-wall CNTs in the oil. In this way, Marquis et al. [71] not only shifted the thermal degradation of the oil to higher temperatures but also enhanced the TC of the base fluid by more than 45% with the use of 1 vol% of single-wall CNTs. The insertion of NPs leads to a more viscous fluid, which is undesirable since higher viscosity is not favored for heat transfer enhancement. Prasher et al. [72] analyzed these opposing effects and concluded that if the rise in viscosity is smaller than the rise in TC by a factor of 4, the use of NFs can help improve heat transfer; otherwise, the disadvantages of NFs overcome their advantages.

In summary, as summarized in Table 3, by using 0.3–4.2% of various NPs, including metals [21], metal oxides [68], CNTs [71], and metal oxides [15] in different shapes, a 16~150% increase in TC of different base fluids was reported. However, there are discrepancies about the impact of different parameters on the final performance of NFs.

### 2.2. Mechanisms

The variation in TC of NFs can be attributed to four major factors: (i) the Brownian motion of NPs; (ii) the molecular layering of the liquid at the NP–liquid interface; (iii) the intrinsic TC properties of the NPs themselves; and (iv) the impact of NP clustering and aggregation [77].

#### 2.2.1. Brownian Motion

There are three particle movements inside the fluid: Brownian motion, resulting from the continuous collisions between fluid molecules and NPs; thermophoretic motion, caused by temperature gradients within the fluid; and osmophoretic motion, arising from concentration gradients within the fluid [78]. Wang et al. [11] studied NFs containing Al_2_O_3_ and CuO NPs in various base fluids. They noted that the heat transferred by particle movements is relatively small compared to diffusion-driven heat transfer. This was supported by low Péclet number (Pe) values that compare advective to diffusive heat transfer rates. Interestingly, while the EMT can predict TC in polymeric composites, it fails for NFs with significant Brownian motion. This suggests that Brownian motion is a crucial factor in the unexpected increase in TC of NFs, highlighting the importance of this phenomenon in the behavior of NF [79].

In general, numerous conflicting reports have been published regarding the impact of Brownian motion. Wen et al. [80] and Ding et al. [46] supported Wang [11] and Keblinski [77] and ignored the role of Brownian motion; in contrast, others emphasized the importance of Brownian motion and held the opposite view [54,63,74,79,81,82,83,84]. In summary, there appears to be no single definitive reason for the increase in TC. Brownian motion could be a contributing factor, effective in some cases but not in others.

#### 2.2.2. Molecular Layering of the Liquid at the NP–Liquid Interface (Kapitza Resistance)

**Figure 6:**
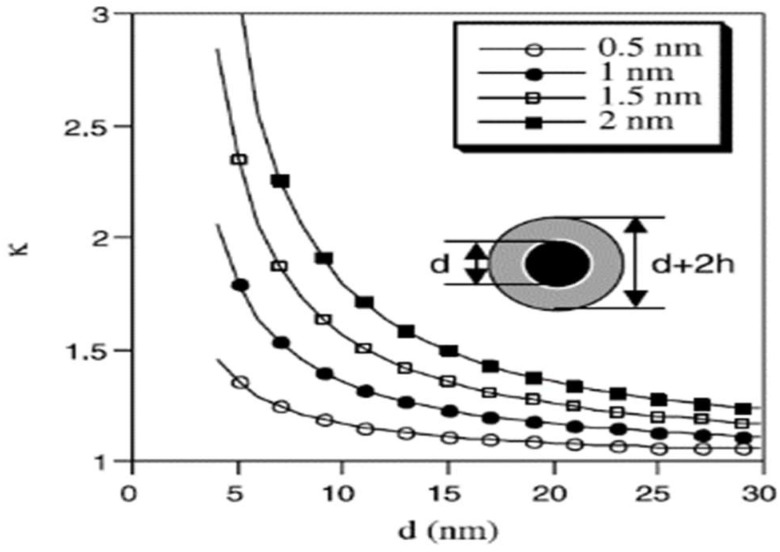
Effect of formation of highly conductive layered-liquid structure on excess TC enhancement (κ) for particles with various diameters [Reproduced with permission from ref. [77]. Copyright 2024, Elsevier].

If the atomic structure of the liquid layer around the solid particles shows a more ordered pattern than the liquid phase, there may be a positive impact on TC [77]. Considering the upper limit of projected outcomes, this layer could be similar to the solid NPs. In that case, the larger effective NP could enhance the TC [77]. Experimental and numerical results have estimated this interfacial layer thickness to be on the order of a few atomic distances, i.e., ~1 nm, for a particle with a diameter of 10 nm. This layer can result in a κ (the ratio of measured TC increase to the increase predicted by the H&C theory) of less than 1.5, which is far lower than many obtained experimental results, showing the inadequacy of this theory for NPs with diameters larger than 10 nm. However, the anomalous increase in TC with NPs smaller than 10 nm in diameter can be justified, as depicted in Figure 6 [77], which signifies the importance of increased specific surface area (SSA). Using the heat conduction equation in spherical coordinates, an expression for calculating the TC of NFs was derived. While the model generated results consistent with some experimental data, it showed limitations in predicting other data accurately. Thus, although the impact of the nanolayer on the system’s TC was shown to be significant, it was suggested that other parameters, such as surface chemistry and inclusion shape, should also be considered. Finally, it was concluded that the nanolayer becomes more important as particle size decreases and nanolayer thickness increases accordingly [85].

#### 2.2.3. Heat Transfer Dynamics Within Nanoparticles

**Figure 7:**
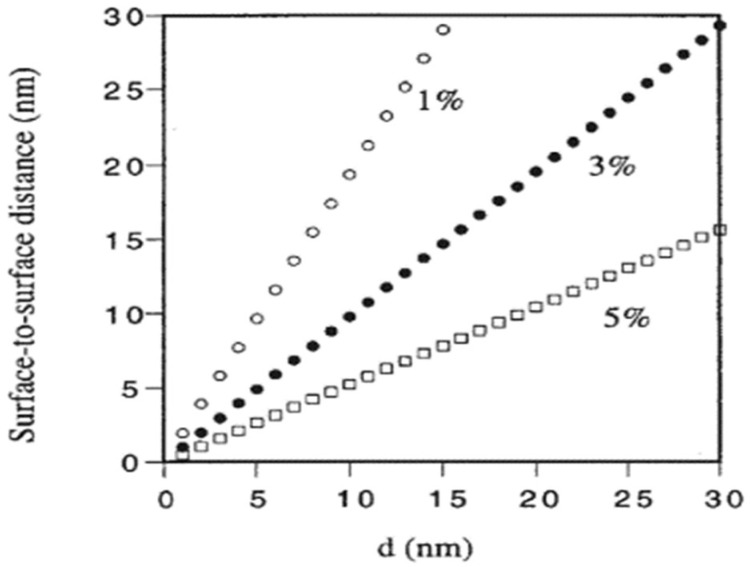
Average surface-to-surface distance between particles for three different particle volume fractions as a function of particle diameter [Reproduced with permission from ref. [77]. Copyright 2024, Elsevier.

In crystalline structures of NPs, heat is transferred by phonons, i.e., propagating lattice vibrations [77]. For typical NPs such as Al_2_O_3_, the mean free path of a phonon is 35 nm at room temperature. Consequently, if d = 10 nm, phonons cannot diffuse within the particle and can only move ballistically across it. Therefore, the diffusion theory used in the H&C model is not applicable. If ballistic phonons can traverse from one NP to another through the liquid phase, a higher TC can be expected [77]. Given that the phonon mean free path in liquids is much shorter than in solids, this transfer will be successful if the NPs are sufficiently close together with a distance of ~2 nm. Figure 7 shows the average surface-to-surface distance between particles for 1, 3, and 5 vol% of NPs as a function of particle diameter, suggesting a feasible phonon transfer [77]. Using different sizes of CuO in the same base fluid, Colangelo et al. [86] measured higher TC for the samples with smaller CuO NPs. To justify this behavior, they relied on improved ballistic phonon transport and reported that ballistic phonon transport is more effective in smaller NPs, resulting in a greater relative TC.

#### 2.2.4. The Effects of Nanoparticle Clustering

**Figure 8:**
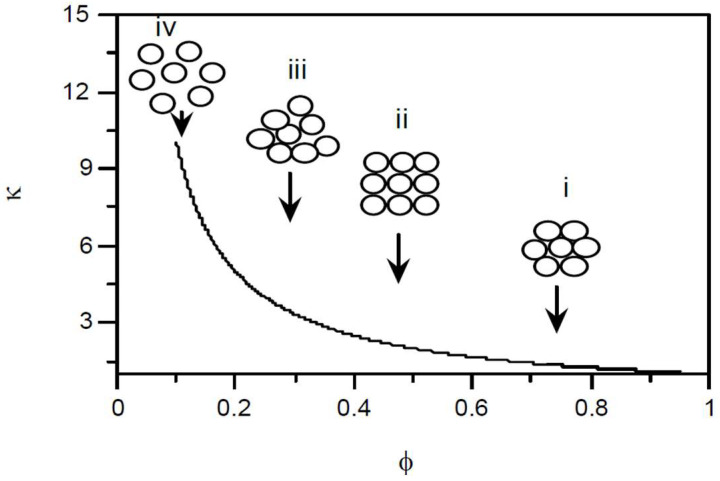
Excess TC ratio (κ) as a function of the packing fraction of the cluster (φ) [Reproduced with permission from ref. [77]. Copyright 2024, Elsevier.

Clustering can be explained using Figure 8, which plots the excess TC ratio (κ) as a function of the packing fraction of the cluster (φ) reducing from (i) to (iv), defined as the ratio of the volume of a single solid particle in the cluster to the total volume of the cluster. Even for closely packed clusters, almost one-fourth of the cluster volume is filled with liquid, resulting in a larger effective volume of highly conductive clusters; however, for loose clusters, the more effective volume becomes available for heat transfer. This localized, particle-rich region facilitates faster heat transfer due to decreased thermal resistance compared to areas with lower particle concentration [77]. Experimental results of a CNTs–water NF reported significant disparities between the experimental data and the predictions of the conventional EMT model at temperatures ranging from 10 to 70 °C. The exclusion of networking and clustering of NPs in EMT macroscopic theory was the major factor for these differences [80].

### 2.3. Factors Affecting the Thermal Conductivity Performance in NFs

#### 2.3.1. Nanoparticle Material

The NP material affects both TC and the stability of NFs. Metals like copper offer high conductivity, metal oxides like CuO provide good stability, while carbon-based materials like graphene offer a balance of high conductivity, flexibility, and low density for heat management. In one of the earliest studies highlighting the importance of NP material, Eastman et al. [73] used CuO and Al_2_O_3_ in water and reported a higher TC enhancement for the CuO-containing NF. In a similar investigation, the effects of Al_2_O_3_ NPs, CuO NPs, and their hybrid were studied in water. Interestingly, despite the higher conductivity of CuO NPs, the addition of Al_2_O_3_ to the CuO solution in the hybrid suspension boosted the TC by 9.8%, even more than the CuO/Water suspension [36]. Another study depicted a faster rate of increase for CuO in comparison to Al_2_O_3_ when NP concentration in water increased [87]. Koo et al. [83] conducted a numerical study on the TC of NFs and indicated the significance of the electric dipole constant. This finding proved the particle type dependence of TC. In another study, although bulk Al_2_O_3_ has higher TC than the bulk of TiO_2_, water-based TiO_2_ showed a higher TC enhancement compared with Al_2_O_3_ [88].

While theoretical mixing theory stresses that NPs with higher TC can potentially improve the overall TC of NFs further in all cases, the practical impact is not always in this direction, probably due to the low concentration of NPs and the overall interaction of other factors.

#### 2.3.2. Nanoparticle Concentration

Increasing NP concentrations in NFs can non-linearly enhance TC due to a higher proportion of thermally conductive solids, formation of NP networks, interfacial fluid layering on NP surfaces, increased Brownian motion, and micro-convection. Various NPs (e.g., MWCNTs [80], Al_2_O_3_ [36,87], and CuO [36,60]) have shown this effect. However, the growth of TC with increasing concentration of NPs does not always exist. Phenomena like viscosity spikes and sedimentation can impair TC increase. For instance, Ceylan et al. [89] found that Ag-Cu alloy NPs in oil reached peak TC enhancement (~35%) at 0.006 vol.%, then dropped due to sedimentation.

**Figure 9:**
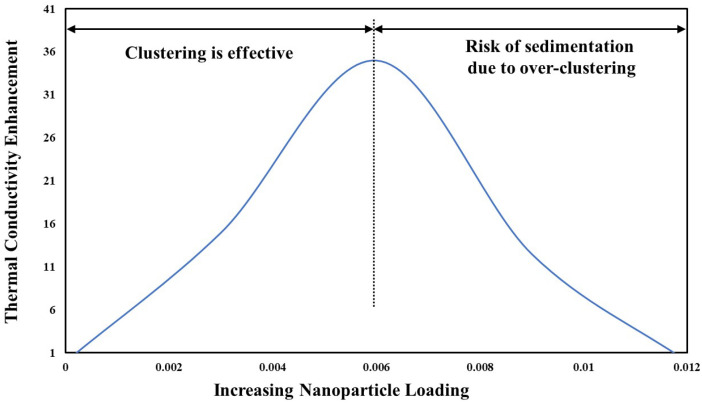
Impact of NP loading on effective TC enhancement of NFs.

In summary, as shown in Figure 9, there seems to be an optimal NP loading for maximum TC enhancement. Below this, particle clustering is insufficient, and a large region of the liquid remains particle-free; above it, over-clustering leads to sedimentation, increased viscosity, and reduced TC. 

#### 2.3.3. Particle Size

The impact of NP size on TC enhancement in NFs can be understood by considering two distinct regimes divided by the phonon mean free path [90]. When NP diameters exceed this threshold, a reduction in size leads to increased TC. This occurs because smaller particles have a larger SSA, which enhances heat transfer at the particle–liquid interface. Conversely, when NP diameters fall below the phonon mean free path, the relationship becomes more complex. In this regime, further increases in SSA cease to be the primary driver of TC enhancement. Instead, phonon scattering at the particle surface becomes dominant, causing decreased TC as the particle size diminishes [20,91].

Bhattacharya et al. [81] used a dynamic simulation approach to evaluate the effective TC of NFs. They demonstrated that for a water–Cu NF, particles with lower diameters exhibit a higher effective TC and attributed this outcome to the pronounced Brownian motion of smaller NPs [81]. In a study on the impact of temperature on TC of water/CuO and water/Al_2_O_3_ NFs, Das et al. [74] observed steeper changes in the water/CuO NF. They ascribed this to the smaller size of CuO NPs, which facilitated better movement and accelerated changes [74]. Colangelo et al. [86] studied different base fluids and Cu NPs with 25, 50, and 100 nm diameters. In all cases, they reported better performance for NPs with smaller diameters and linked this behavior to ballistic phonon transport, as was discussed by Keblinski [77]. Opposingly, Timofeeva et al. [92] studied SiC–water NF and reported an increase in TC with increasing the particle diameter from 20 nm to 90 nm. They related this finding to the increased thickness of the base fluid layer around smaller NPs, which lacked thermal transport.

#### 2.3.4. Particle Shape

**Figure 10:**
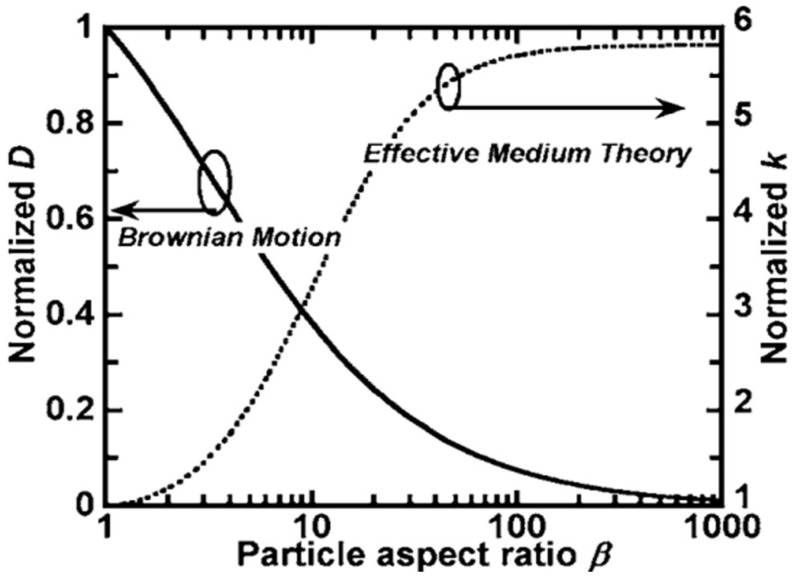
Reliance of normalized Brownian diffusivity and normalized TC of NFs estimated by EMT on β [Reproduced with permission from ref. [16]. Copyright 2024, AIP Publishing].

NPs with dissimilar shapes can bring about various impacts on the TC of the final NF [16], and by moving from spherical geometry to elongated and rod-shaped particles, NFs with higher TC can be obtained [20]. Xie et al. [15] used two different shapes of SiC NPs, spherical and cylindrical, in water and EG. Confirming the H&C model, they showed that the NF containing the cylindrical SiC NPs has a higher TC [15]. Two different types of TiO_2_ NPs in spherical and cylindrical shapes were synthesized, and NPs with a cylindrical shape outperformed spherical ones in improving the TC of NFs, which highlights the importance of NP shape [93]. The same result was also obtained during solidification of water–TiO_2_ NF [39]. Yang et al. [16] dispersed cylindrical Bi_2_Te_3_ NPs in perfluoro-n-hexane and hexadecane oil base fluids. Contrary to Patel [82], they concluded that the TC of NFs decreases with rising temperature and this discrepancy was attributed to the particle aspect ratio (β), the ratio of the major axis to the minor axis. Patel [82] used spherical particles (low β), while Yang et al. [16] used cylindrical NPs with a higher β. The aspect ratio is crucial because, for spherical particles, Brownian motion is a strong heat transfer mechanism. However, as can be seen in Figure 10, with an increasing β and deviation from spherical geometry, Brownian motion’s importance decreases due to drag forces, making diffusion conduction more significant [16].

Brownian diffusivity, D, considers the role of NPs as “heat boats” in the direct transfer of energy and its contribution to improving the TC. Both Brownian and conduction diffusivities are normalized to spherical geometry. As the aspect ratio increases, conduction diffusivity surpasses Brownian diffusivity, reducing TC’s temperature dependency (as seen in the case of Patel [82]). Thus, all geometries show distinct contributions from both mechanisms [16]. Ghosh et al.’s [94] molecular dynamics (MDs) study depicted that particle shape and fluid temperature impact NF heat transfer. Cylindrical NPs, with a higher β, exchange heat more effectively than spherical NPs. Generally, in a constant volume, the surface area of different shapes varies according to the following order: spheres < cylinders < cubes. More specifically, for cylinders, the surface area increases with increasing β [20,94].

Colangelo et al. [86] formulated NFs using water and diathermic oil with particles of different shapes and materials, including spherical CuO and Al_2_O_3_ and elongated ZnO. Despite ZnO’s lower inherent TC compared to CuO and Al_2_O_3_, ZnO-based NFs outperformed the others and exhibited the highest TC enhancement.

To summarize, although Lee et al. [61] showed that size has a bigger effect than shape on TC enhancement of NFs, NP shape also has a great impact. The better performance of NPs with elongated shapes stems from the larger surface area and modified heat transfer mechanisms associated with non-spherical shapes.

#### 2.3.5. Nanofluid Stability and Dispersion

The effective use of NFs relies on maintaining colloidal stability to prevent NP agglomeration, primarily caused by van der Waals forces [20]. Sonication and chemical techniques like surfactants and pH adjustment improve stability [95,96]. Surfactants prevent NP agglomeration but may reduce TC at high concentrations. Both ionic and non-ionic surfactants stabilize NFs through electrostatic repulsion or steric hindrance. A study comparing Disperbyk-190 and Redline Water Wetter in a water-based NF with BaTiO_3_, ITO, and β-SiC NPs showed both provided good stability, but Redline Water Wetter allowed easier restoration of stability after settling [97]. Another study found that 0.1% SDBS increased TC by 10.4% in a suspension of 30% alkyl hydrocarbon and 1% TiO_2_ due to enhanced stability [52].

pH also influences NP stability, enhancing TC when far from the isoelectric point. Xie et al. [91] demonstrated the effect of pH modification ranging from 11.5 to 2 on maintaining NPs fully suspended in the base fluid without agglomerations and proved an increase in relative TC as the pH moved further away from the isoelectric point [91,98].

Sonication helps disperse NPs, with effectiveness dependent on time, frequency, and concentration, balancing improved TC and viscosity control [78]. Surfactant-free ZnO-EG NF was used to study the effect of sonication time on the relative TC. Meanwhile, DLS was used to measure the particle diameter. The maximum improvement in TC was observed after 60 h of sonication, corresponding to the smallest particle size in the suspension [99]. In other similar research, TC and viscosity of water–Al_2_O_3_ NFs at various Al_2_O_3_ concentrations (0.5–20 vol.%) and sonication times were investigated [100]. The study found a 16.1% TC improvement at 2 vol.% Al_2_O_3_ after 2 h of sonication, but longer sonication reduced TC. Higher Al_2_O_3_ concentrations needed more sonication for uniform dispersion. At 1.5 vol.% Al_2_O_3_, viscosity dropped by 33% with increased sonication time, then rose by 13% due to NP re-agglomeration [100].

In general, while controlled aggregation and clustering can create additional conduction paths that enhance TC, excessive aggregation and clustering may also increase viscosity and cause settling, which are undesirable outcomes. Therefore, a combination of physical and chemical methods is usually necessary to achieve optimal NF stability and dispersion for practical applications.

#### 2.3.6. Temperature

An increase in temperature raises the kinetic energy of the NP in the NF, which intensifies Brownian motion and micro-convection and enhances particle–particle and particle–liquid interactions [78]. Additionally, increasing temperature causes a drop in viscosity, which favors TC [101]. Finally, increasing temperature can bring about higher TC by increasing the interfacial layer thickness and the SSA [20,102]. However, increasing temperature may negate the NF stability, which in turn can diminish the TC [78,80].

The temperature dependence of TC enhancement was studied for two water-based NFs containing Al_2_O_3_ and CuO NPs [36,74], and the TC enhancement increased with temperature for both NFs. For Al_2_O_3_ NFs, the enhancement grew from 2% at 21 °C to 10.8% at 51 °C and for CuO NFs, it increased from 6.5% to 29% over the same temperature range [74]. Hu et al. [50] dispersed AlN NPs in ethanol. At constant NP concentration, TC remained unchanged at 0 °C and 20 °C due to opposing effects of temperature on agglomeration and Brownian motion. At lower NP concentrations, higher temperatures enhanced Brownian motion and TC. However, at higher temperatures, agglomeration reduced TC [50]. Sundar et al. [103] synthesized a novel MWCNT-Fe_3_O_4_ nanocomposite powder using an in situ method and dispersed it in water. At 20 °C and 60 °C, the addition of only 0.3% of the nanocomposite increased the TC by 13.88% and 28.46%, respectively, compared to the base fluid at the same temperatures, suggesting that NFs can provide higher cooling rates where required [74].

## 3. Enhancing Specific Heat Capacity of Nanofluids

**Figure 11:**
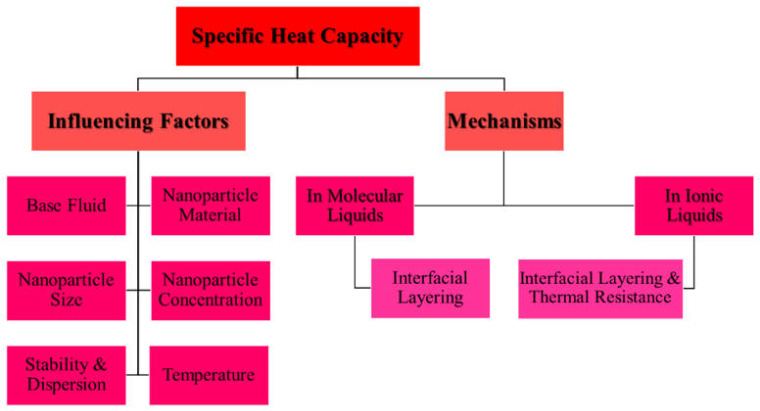
Mechanisms involved in SHC enhancement of NFs and the influencing factors.

The SHC of an NF represents the amount of heat required to raise the temperature of a unit mass of NF by one degree Kelvin and quantifies therefore its ability to store thermal energy. Enhanced SHC improves heat transfer in two ways. Firstly, since a higher SHC increases the Prandtl number (Pr) linearly, and Pr directly influences the Nusselt number (Nu), this leads to an overall enhancement in heat transfer coefficients [104]. Secondly, it allows fluids to absorb or release heat with smaller temperature changes, maintaining a higher mean temperature difference between hot and cold streams. This contributes to more efficient heat exchange, potentially reducing heat exchanger size. Although most studies report that metal, metal oxide, and carbonaceous NPs decrease the SHC of NFs, the mechanisms behind these changes seem to be more complex. The mechanisms involved and the parameters influencing SHC enhancements are summarized in Figure 11.

### 3.1. Review on Specific Heat Capacity of Nanofluids

The changes in SHC of NFs vary significantly depending on the base fluid, as different underlying mechanisms come into play. Among various base fluids, molten salts have shown notable SHC increases attributed to their ionic nature. Besides the base fluid, the type, size, and concentration of NPs also significantly affect the SHC.

#### 3.1.1. Molecular Liquids

Ravikanth et al. [105] used Al_2_O_3_ NPs to prepare a water/EG-based NF, and the SHC of the NF decreased by increasing the NP content. The reason for this is attributed to the lower SHC of the Al_2_O_3_ NPs compared to the base fluid [105]. The same results were also obtained by Barbes et al. [60] for a water/EG-based NF containing CuO NPs. Goldenstein et al. [97] formulated a water–BaTiO_3_ NF and found decreasing SHC with increasing BaTiO_3_. The use of MgO, ZnO, and ZrO_2_ NPs in a mixture of EG and water was evaluated, and the reduction in SHC for all NFs was observed with an increase in the volume fraction of NPs. The volumetric SHC (the amount of heat required to raise the temperature of a unit volume of NF by one degree Kelvin) was also determined [106], and in contrast to SHC, volumetric SHC remained constant or slightly increased after the addition of NPs [106]. Three suspensions of Pd nanoplate in Dowtherm (6%, 3%, and 1.2%) were prepared, and all samples showed higher SHC than the base fluid [19].

Currently, the results are mixed. While the majority of studies report a negative impact of NPs on the SHC of molecular fluids, some findings show the opposite effect. Therefore, a detailed discussion of the possible mechanisms affecting the SHC of NFs is essential.

#### 3.1.2. Ionic Liquids

Ionic liquids (ILs) are salts (with no additional solvent) that possess melting points below 100 °C [48]. ILs are generally composed of an organic cation coupled with an inorganic or organic anion. The low melting points arise because one or both of the ions are bulky and have low molecular symmetry [48]. The use of bulky, asymmetric ions frustrates the packing of ions into a lattice structure, decreasing the lattice energy and lowering the melting point [48]. Cherches et al. [107] studied the use of Al_2_O_3_ NPs in [C4mim][BF4]. They observed that at low concentrations, NPs had no impact on SHC. However, at higher concentrations, Al_2_O_3_ NPs decreased the SHC of the formulated NF by 8%, attributed to the low SHC of Al_2_O_3_ NPs. In a later study, Cherches et al. [108] suspended MWCNTs instead of Al_2_O_3_ NPs. At low concentrations (0.025 and 0.05 mass%), they observed up to a 4% increase in SHC. However, with increasing MWCNT fraction, the SHC of the formulated NF dropped by 6% compared to the base fluid. These results differ from their previous findings [107], which reported a monotonous decrease in SHC with increasing mass content of Al_2_O_3_ in [C4mim][BF4] ionic liquid. Altun et al. [35] examined the SHC of [EMIM][EtSO_4_]-based NF with Al_2_O_3_ NPs. They concluded that 0.96 vol.% Al_2_O_3_ NPs improved SHC over pure ionic fluid, but higher concentrations decreased it.

To conclude, the SHC of formulated ionic fluids appears to be influenced by multiple factors, including the specific type of ionic liquid, concentration of NPs, and characteristics of the NPs and their particular combination with ionic liquid.

#### 3.1.3. Molten Salts

Molten salts, like ILs, consist of ions but with similar sizes. Due to their high boiling point and stability, they can be effectively utilized in high temperatures without any rise in pressure. Despite these advantages, their SHC and TC are quite low (SHC < 2 kJ/kg°C, TC < 1 W/mK) [109]. Therefore, numerous efforts have been made to improve the SHC of molten salts [24,45,109,110,111] by the incorporation of NPs, and significant enhancements are being made. To maximize the SHC of molten Hitec salt using NPs, the volume fraction of Al_2_O_3_ was optimized. The addition of alumina NPs did not have a consistent impact on the SHC. Initially, by increasing the NPs, the SHC rose but then fell below the SHC of the pure molten salt [44]. Kwak et al. [24] inserted 2.5 mass% SiO_2_ NPs in metal salt eutectic and obtained a 14.59% enhancement in SHC. Shin and Banerjee [110] aimed to amend the SHC of a eutectic chloride salt using TiO_2_ NPs. In the solid eutectic chloride salt, the SHC was invariant with rising temperature. However, in the liquid phase, the SHC improved with increasing temperature. In both states, the SHC of the NF surpassed the base fluid [110].

In conclusion, the evidence suggests that incorporating NPs into molten salts generally enhances SHC, regardless of the specific type of NP used.

### 3.2. Mechanisms

The irregular variations in SHC with NP concentration challenge the predictions of mixing theory, which suggests a decrease in SHC due to the inherently lower SHC of NPs compared to base fluids. However, some studies report contrasting findings, highlighting the presence of complex underlying mechanisms.

#### 3.2.1. In Molecular Liquids

When an NP is suspended inside a fluid, as described by Hentschke’s “interacting meso-layer model” [90], thick layers of fluid molecules (~100 nm) form around NPs with slightly higher SHC than the bulk liquid. As NP concentration increases, SHC initially rises, peaks, and then decreases when these layers overlap, forming more solid-like structures that reduce SHC. Carrillo-Berdugo et al. [19] demonstrated that optimal concentrations of Pd nanoplates in Dowtherm enhance SHC through strong interfacial layering, which can be further improved by promoting chemisorption over physisorption [112]. Jin et al. [113] used MD simulations to study water–Cu NFs in three configurations: confined, sessile, and freestanding films. All NF films showed higher SHC than water films of the same thickness, with confined films achieving the highest SHC due to stronger molecular structuring near solid surfaces. Sessile films exhibited intermediate SHC values due to asymmetry in density distribution, while freestanding films had the lowest SHC. These results highlight the critical role of interfacial effects in influencing SHC.

#### 3.2.2. In Molten Salts

**Figure 12:**
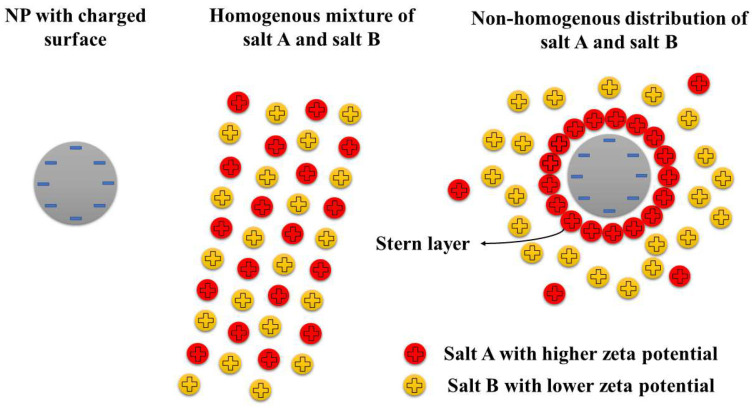
Microstructure formation resulting from variations in the zeta potential of different salts.

In molten salts, as in molecular liquids, semi-solid layers form around the NPs and play a crucial role. These compressed layers enhance thermal energy storage capacity [45] by requiring additional energy to break down their structure [114], effectively serving as an extra mechanism for heat retention. These findings align with results from Wang et al. [115] and Chieruzzi et al. [111], emphasizing that the high surface energy of NPs with large surface areas is crucial for SHC enhancement. However, surface area enlargement has more complexity. Another reason for surface enhancement can be attributed to the microstructure formation due to electrostatic interaction [116]. The EDL has three key components: First, the surface charge is determined by the core particle itself and creates the surface potential. Second, the Stern layer contains tightly bound counterions attracted to the core, ending at what is called the Stern potential. Third, the diffuse layer forms an interactive boundary between the particle and surrounding medium, containing exchangeable counterions and defining the particle’s zeta potential. For oxide materials like silica or alumina, surface hydroxyl groups create a negative charge. In binary salt systems containing alkali metals (K^+^, Na^+^, and Li^+^) and complex anions (NO_3_^−^ or CO_3_^2−^), the different salts will have varying zeta potentials despite the consistent positive charge of the metals. This difference causes preferential attraction; the salt with higher zeta potential accumulates around negatively charged NPs while simultaneously repelling the other salt species [116]. This process is clearly illustrated in Figure 12. Jeong and Jo [117] used zeta potential measurements to analyze the Stern layer and Debye length in KNO_3_-SiO_2_ NFs, identifying the EDL as the primary factor for SHC enhancement. Yuan et al. [118] showed that higher NP charges lead to denser interfacial layers, improving SHC in Li_2_CO_3_/K_2_CO_3_-SWCNT NFs. Jo and Banerjee’s MD simulations revealed that variations in local density and ion composition in the compressed ion layer influence the level of SHC enhancement [119]. Tiznobaic and Shin [120] observed that charged SiO_2_ NPs altered the local chemistry of the surrounding fluid, forming needle-like substructures and affecting SHC. Shin et al. [121] highlighted the role of fractal structures and increased NP surface area in enhancing SHC when metallic NPs are added to molten salts. Chieruzzi et al. [111,122] showed that even without substructures, SHC increases due to smaller crystal sizes in NP-doped molten salts. Jeong and Jo [117] noted that reduced crystallite sizes and enlarged surface areas in KNO_3_-SiO_2_ NFs contributed to SHC improvements.

In molten salts, these mechanisms are complemented by compositional and textural changes, highlighting the critical role of NP properties and system-specific dynamics in improving SHC.

### 3.3. Factors Affecting the Specific Heat Capacity Performance in NFs

#### 3.3.1. Base Fluid

SHC of NFs depends not only on NP type and preparation methods but also significantly on the base fluid. While NP-doped molten salts consistently show promising performance [19,23,123,124,125], for molecular liquids, it depends on the difference between the SHC of the NP and the base fluid [10,37,60]. This varying behavior may stem from different heat transfer mechanisms in various base fluids, as well as distinct interactions between the base fluid and NPs.

#### 3.3.2. Nanoparticle Material

Different NPs exhibit varying effects on SHC when added to the same base fluids. For instance, MgO, ZnO, and ZrO_2_ caused different reductions in SHC when added to a water–ethylene glycol mixture [106]. In ILs, Oster et al. [126] found that CNT, BN, and graphite increased SHC by 5–13%, 5–21%, and 12–28%, respectively, suggesting that SHC enhancement depends more on NP type than base fluid. Cherches et al. [107,108] observed different SHC behaviors when using Al_2_O_3_ and MWCNT in [C4mim][BF4]. In other research conducted by Sang and Liu [32], SiO_2_, CuO, TiO_2_, and Al_2_O_3_ NPs were used to enhance the SHC of a ternary carbonates NF. Despite the higher SHC of Al_2_O_3_ compared to the other particles, SiO_2_-doped NF presented the highest SHC.

In conclusion, while several reports confirm the importance of NP material, using components with higher SHC does not necessarily result in an NF with higher overall SHC.

#### 3.3.3. Nanoparticle Concentration

The varying concentrations of NPs can change the thermal properties of the NFs; however, the direction of these changes is not the same in all cases. While in the major part of the reports, SHC decreases with increasing NP concentration [60,107,127], Lasfargues et al. [128] concluded different concentration-dependent behaviors for dispersed TiO_2_ and CuO in molten salt; CuO showed maximum enhancement at its lowest concentration, whereas TiO_2_ demonstrated no concentration dependency.

As suggested by the interacting meso-layer theory, there seems to be an optimal NP concentration at which the SHC of the NF reaches a peak, after which it declines.

#### 3.3.4. Particle Size

There are not many conclusive publications on the impact of particle size on the SHC of formulated NF. Lu and Huang [129] studied a molten salt mixture of NaNO_3_/KNO_3_ doped with alumina NPs of two different sizes. Their key finding was that the SHC was higher for the NF containing the larger NPs. A similar result was reported by Dudda and Shin [130] in their study of the same NaNO_3_/KNO_3_ molten salt mixture doped with 1 mass% SiO_2_ NPs. In contrast, Tiznobaik and Shin [120] did not find any influence of particle size in their investigation of a Li_2_CO_3_/K_2_CO_3_ eutectic molten salt doped with SiO_2_ NPs of 5 to 60 nm in diameter. In another study, 5, 10, 30, and 60 nm NPs were suspended in a ternary carbonate fluid [32]. While the size of NPs was below 30 nm, SHC increased with increasing the size of NPs. Over 30 nm, the SHC decreased with increasing the NP size. Honarkhah et al. [131] examined how NP size influences the SHC of water-based nanofluids in nanochannels. They discovered that the SHC of NFs increases as the size of the NPs grows.

Limited research suggests that larger NPs may further improve the SHC of NFs, provided their suspension remains stable.

#### 3.3.5. Nanofluid Stability and Dispersion

Similar to TC, the stability of NPs suspension significantly affects the SHC of NFs. Studies by Byeongnam and Banerjee [119,132] show that better stabilization of MWCNT and graphite NPs in eutectic salts led to higher SHC. Chieruzzi et al. [122] found that the SHC of NFs with oxide NPs in nitrate salt mixtures depends on the mixing method, with smaller aggregates yielding higher SHC improvements. The effects of different surfactants, Sodium SDS—GA and SDBS, were examined in a suspension of CNTs in a ternary fluid. Among various samples, the one with SDBS exhibited the most significant improvement in SHC by 78.3% [125]. Although the use of surfactant alone is incapable of augmenting the SHC, its contribution to better suspension of CNTs could further help elevate the SHC [125]. However, Ali’s [133] study on graphite–water NFs demonstrated that while higher surfactant content increased stability, it decreased SHC remarkably.

In conclusion, despite limited research, it appears that higher dispersion and stability generally amplify the NPs’ impact on SHC, whether positive or negative. When NPs improve SHC, better stability enhances this effect; conversely, when NPs decrease SHC, higher stability exacerbates this reduction.

#### 3.3.6. Temperature

All known studies report increased SHC of NFs with temperature, though the rate varies, likely due to base fluid and NP types [107,128,129,130,134,135]. For instance, kerosene-Fe_3_O_4_ NF showed a slower SHC increase than pure kerosene [135], while molten salt–SiO_2_ NF exhibited steeper changes than their base fluid [130]. This temperature dependency can be attributed to the fact that particle vibrations remain in the ground state at lower temperatures and require higher temperatures to overcome this state. As thermal energy increases, more degrees of freedom in the particles are unlocked, enabling greater energy storage [136].

## 4. Enhancing Convective Heat Transfer of Nanofluids

Natural and forced convection have crucial impacts on heat transfer in fluids. Natural convection relies on buoyancy-driven fluid motions, while forced convection needs external forces provided by pumps. If a liquid’s vapor pressure exceeds the pressure exerted on its surface, boiling can occur, transferring significant thermal energy. In the following part, the impacts of NPs in these processes are discussed.

### 4.1. Effects of NPs on Natural Convection

Natural convection is advantageous due to its spontaneity and lack of reliance on mechanical systems, which eliminates the associated risks of mechanical and electrical failures, reduces costs, and enhances device reliability. In addition, it minimizes acoustic and electromagnetic noises by having no moving parts, making it attractive for heat transfer in confined spaces common in various industrial applications [137].

To study the impact of NPs on natural convection, water-based NFs of CuO and Al_2_O_3_ NPs were investigated in a horizontal cylinder, heated from one side and cooled from the other side. Results indicated that although the NPs could increase the thermal homogeneity of the fluid, they had a destructive impact on natural convection [138]. Later, the same unexpected decrease in natural convection was also observed for a water–TiO_2_ NF and increased viscosity was recognized as the main reason [139]. Ag-TiO_2_ NPs were synthesized to utilize the simultaneous effect of two elements in an NF [137]. This NF showed increased TC with higher temperature and NP concentration, outperforming an NF containing TiO_2_ NPs. Tested in a cubic cavity with varying heat input, the NF’s convective heat transfer coefficient reached a maximum at 0.15 vol% NP concentration before dropping, but it always surpassed the base fluid’s natural convection [137]. Nnanna et al. [140] explored the natural convection behavior of an Al_2_O_3_–distilled water NF and found an enhancement in heat transfer at low NP concentration (0.2~2.1 vol%) due to the increased thermal diffusion.

NPs have dual effects on natural convection: increased viscosity hinders it, while enhanced TC improve it. Consequently, an optimal point likely exists where the interactions between these factors maximize natural convection.

### 4.2. Effects of NPs on Forced Convection

Forced convection is the most frequent heat transfer approach with widespread industrial applications. Similar to natural convection, the interactions between enhanced TC and elevated viscosity can specify the overall heat transfer efficiency. Li and Xuan [141,142] found that the addition of 2 vol% Cu NPs in water improved the heat transfer coefficient by 60% without a significant pressure drop. The chaotic behavior of NPs flattened the temperature gradient in NF, increasing the temperature difference between the tube wall and fluid as the driving force, thus enhancing heat transfer [141,142].

When a suspension flows inside a tube, particles may accumulate in the axial region due to induced shear by varying radial velocity. Conversely, Brownian diffusion tries to maintain equilibrium, as described by Pe et al. [143]. Thermal properties of NFs were studied using a model considering particle migration due to gradients in viscosity, shear rate, and Brownian motion. This migration caused significant non-homogeneity in particle distribution, especially for large particles at high concentrations, impacting local TC. Axial regions with more NP accumulations had higher TC than regions near the wall. They concluded that increasing Reynolds (Re) increases the Pe and non-homogeneity, resulting in varying TC across the tube cross-section and increased Nu [143,144]. Regarding the variations of the heat transfer coefficient along the axial direction, Ding et al. [144] studied CNTs–water NFs and observed a decrease in the heat transfer coefficient, with the maximum heat transfer rate occurring in the entrance region. As a complementary note, at a certain distance from the pipe entrance, the boundary layer (the region where the fluid velocity transitions from zero at the wall to the maximum at the pipe center) continues to grow until it stabilizes. Once this occurs, the velocity profile becomes fully developed and remains unchanged along the flow direction. This condition is known as “developed flow”. The heat transfer coefficient depends on two main factors—the TC of the fluid and the thickness of the boundary layer—and is calculated as the ratio among both. Therefore, it reaches its maximum in the entrance region, where the thickness of the boundary layer is minimal [46], confirmed by a numerical study conducted by Balla et al. [145]. Likewise, Ho et al. [146] stated that the decrease in the heat transfer coefficient along the flow direction is due to the reduced SHC of NFs. Tumuluri et al. [147] studied the simultaneous use of MWCNT and microencapsulated octane as the PCM. Although the hybrid NF outperformed at constant Re, no significant advantage was seen at constant velocity and pumping power. Achieving the same Re in NFs requires more energy, impeding the system’s overall efficiency [147,148]. Similar findings were also reported by Suresh et al. [149] and Ho et al. [146] for Al_2_O_3_-Cu/water and Al_2_O_3_-microencapsulated PCM (MEPCM)/water NFs, respectively.

In summary, NPs enhance forced convection by improving TC, inducing Brownian motion, creating non-homogeneity, and increasing driving force. However, as summarized in Table 4, the resulting increased flow resistance may raise operational costs, potentially limiting NFs’ viability in large-scale applications.

### 4.3. Effects of NPs on Boiling

Boiling is a phase change process where vapor bubbles form in a liquid when its vapor pressure exceeds the surrounding pressure. NPs can significantly influence boiling by altering surface characteristics, enhancing TC and modifying bubble dynamics. These effects can increase heat flux and the heat transfer coefficient, though the exact impact depends on various factors like NP type, NP concentration, base fluid properties, and NF viscosity.

Trisaksri et al. [30] studied the use of TiO_2_ NPs in an R141b-based domestic refrigerator. This study suggested that by shifting the pool boiling to higher temperatures and reducing the heat transfer coefficient, the NPs had a negative impact on the pool boiling of R141b in the evaporator of the refrigerator. Park et al. [157] evaluated the effects of Al_2_O_3_–water NFs with varying Al_2_O_3_ NP concentrations on quenching a heated stainless steel sphere. Results depicted that NPs decreased film boiling and cooling rate during quenching. NPs were believed to stabilize the vapor film around themselves, causing reduced heat transfer. An unwashed sphere with loosely attached NPs did not form a stable vapor film, resulting in rapid cooling [158]. Likewise, Khandekar et al. [159] described how NFs change the performance of a gravity-assisted two-phase thermosyphon. They found that the interaction of NPs with nucleation cavities is more imperative than the increase in TC for pool boiling behavior. The heating surface’s roughness was measured before and after pool boiling, showing a significant reduction. They concluded that NPs trapped in the nucleation cavities reduced their size and active nucleation site density. In another study, Noie et al. [160] studied a water–Al_2_O_3_ NF in a thermosyphon. They found that adding more NPs improved efficiency and heat transfer. The system operated closer to an isothermal condition and had a lower maximum wall temperature, indicating better performance [160]. This isothermal behavior was also reported by Chien et al. [161], who studied a gold–water NF in a heat pipe.

In general, heat pipes transfer heat by using a heat carrier fluid that evaporates at the heat source and condenses at the heat sink. The vapor moves to the heat sink, releases heat, and returns as a liquid to the heat source via capillary forces or gravity, and depending on the mechanism applied, steam and liquid may move concurrently or counter-currently. Ma et al. [162] used diamond–water NF in an oscillating heat pipe, reducing thermal resistance and improving heat transfer by a factor of 3.4 at 70 °C. The NF was unstable, with NPs settling when the liquid was still. However, heat added to the evaporator caused strong liquid movements, which kept the NPs suspended. In other research, specimens with Al_2_O_3_ NPs, MWCNT, and a hybrid of Al_2_O_3_ NPs and MWCNT were examined in an oscillating heat pipe. Among all the prepared samples, the hybrid NFs exhibited the lowest resistance, while the NF containing MWCNT demonstrated the highest resistance due to its high viscosity [163].

In boiling, viscosity and surface characteristics are crucial factors. While higher surface roughness facilitates bubble formation and heat transfer, excessive viscosity impedes these processes. Furthermore, the formation of NP deposits on heat transfer surfaces may decrease the surface roughness, impairing the boiling accordingly.

A summary of the impact of NFs on heat transfer enhancement is provided in Table 5.

## 5. Combined Effects of Nanoparticles on the Thermal Conductivity and Heat Capacity of Nanofluids

**Figure 13:**
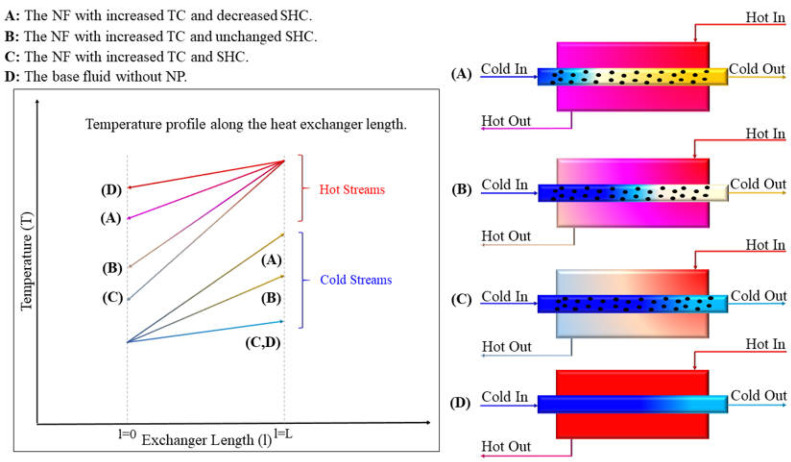
Scheme of the temperature profile of a heat exchanger depending on the thermal properties of the NF: (**A**) the NF with increased TC and decreased SHC, a good heat transfer but with great changes in temperature of the cooling fluid; (**B**) the NF with increased TC and unchanged SHC, a better heat transfer with lower changes in temperature of the cooling fluid; (**C**) the NF with increased TC and SHC, the best heat transfer with limited changes in temperature of the cooling fluid; and (**D**) the base fluid without NP, the worst heat transfer with insufficient cooling of hot stream.

Most research on NP-enhanced HTFs has focused primarily on improving either TC or SHC. Often, changes in other thermal properties are overlooked or insufficiently discussed or yield suboptimal results. However, the importance of SHC and TC can be ignored. Although TC is considered the most influential parameter for heating and cooling applications, decreased SHC can result in a poor contribution to the heat transfer coefficient and the loss of heat transfer capacity. In Figure 13, four different cases are shown comparatively where, in (A), the NF has improved TC but decreased SHC; in (B), the NF has improved TC, but SHC remained unchanged (SHC of the NF is assumed the same as the base fluid); in (C), the NF has improved TC and SHC; and finally, case (D) represents the base fluid without any manipulation. While the original base fluid fails to cool the hot stream due to its poor thermal properties, an NF with high TC but low SHC can cool the hot stream. However, this results in a significant increase in the NF’s temperature as a penalty (case A). In practical cooling applications, excessively high temperatures of the returning cooling medium can lead to operational issues. For instance, in cooling towers, elevated temperatures accelerate water evaporation and loss, impairing system efficiency. By improving the SHC, case (C), the heat transfer improves, and both streams show lower temperatures. Additionally, the use of heat carriers with improved SHC and TC can greatly benefit processes that require isothermal heating, such as the food and pharmaceutical industries. Lastly, as is shown in the diagram of Figure 13, with increasing TC and SHC, the temperature approach rises, leading to a smaller heat exchanger. Therefore, this review considers simultaneous changes in both parameters and discusses various cases for the first time. A summary of the results obtained in some papers, where both TC and SHC were measured, is reported in Table 6.

### 5.1. In Nanofluids

When NPs that do not undergo phase changes are added to base fluids, the resulting NF typically experiences a decrease in SHC and an increase in TC, except for molten salts, where typically, both parameters rise. He et al. [54] suspended spherical TiO_2_ in a 22.5% BaCl_2_ solution. With 1.13 vol% TiO_2_ at −5 °C and 25 °C, TC increased by 12.76% and 16.74%, respectively. However, the SHC was reduced by 12.4% at 10 °C. Additionally, TiO_2_ NPs raised viscosity by 31.9%, making the particle suspension easier but reducing the pumpablility [54]. Saeedinia et al. [53] used 2 mass% CuO NP in motor oil and witnessed an increase of 6.2% in TC and a 23% decrease in SHC compared to the oil. Barbes et al. [104] formulated an NF composed of Al_2_O_3_ NPs stabilized in a mixture of water and glycol. The relative TC improved with the rising NP concentration, while the SHC consistently reduced as the NP content increased [104]. The same result was reported by Jafari when they added Al_2_O_3_ NPs with a 20 nm diameter to the water [37]. Selvam et al. [10] used graphene nanoplatelets in a mixture of water and EG. With increasing NP concentration in the base fluid, TC increased to 18% at the highest NP concentration of ~0.45 vol%. Similar increasing and decreasing behaviors for TC and SHC were also reported by Ali [133] for water–graphene NF. Another study on water-based NFs containing equal amounts of Al_2_O_3_ and CNT NPs showed mixed results [34]. Despite promising enhancements in SHC and TC, viscosity also increased by 100% [34] unwantedly. Rodriguez et al. [27] used graphene NPs in DMAc. TC increased with NP addition, peaking at a 48% enhancement in 0.18 mass% DMAc NF, and SHC increased by 18% in 0.11 mass% NP DMAc NF. Teruel et al. [23] formulated MoSe_2_ nanosheet–Dowtherm NF and reported a 7.2% increase in SHC and a 15% increase in TC as the best results without a significant rise in viscosity. In a study by Morino et al. [124], 0.1 mass% of WSe_2_ nanosheets were dispersed in the base fluid. This NF exhibited a 4.7% enhancement in SHC. The TC showed variable increases, with the maximum improvement reaching 64%. Sang et al. [125] used CNTs to modify the thermal properties of molten salt. While increasing CNTs consistently improved thermal diffusivity in all cases, the most significant improvements of 149.2% and 78.3% were measured for TC and SHC, respectively. In another study [166], the impact of Al_2_O_3_ NPs on a molten salt composed of equal percentages of NaCl and KCl was investigated using molecular simulation, and results showed a concurrent increase in SHC and TC of 14.9% and 10%, respectively [166]. Das et al. [168] examined how amine-functionalized graphene oxide NPs (GO-NH2 NPs) affect a deep eutectic solvent and reported a rise of 55.28% in heat transfer coefficient for an NF containing only 1.01 vol.% of NPs. Additionally, the formulated NF had a higher SHC than the base fluid due to the higher SHC of the GO-NH2 NPs [168]. Three NFs containing 0.0005, 0.001, and 0.002 mass% Pd NPs in silicon were formulated. At high concentrations, SHC of the NF increased with increasing NP and hit a peak at 5.5%. Regarding TC, the best improvement was made at the highest temperature, 100 °C, and the most concentrated NF showed an 8.5% increase in TC [28]. A stable NF containing 0.01 vol% Pd and Au NPs was formulated using a eutectic and azeotropic mixture of biphenyl/diphenyl oxides as the base fluid. The addition of Pd and Au NPs enhanced the SHC of the base fluid by 12.9% and 12%, respectively, and improved TC by 11.5% and 24.9%, respectively, without negatively affecting the fluid’s rheological properties [123].

### 5.2. In Phase Change Slurries

A phase change slurry (PCS) is a class of HTFs consisting of a base fluid and dispersed PCMs, which usually have an increased heat capacity and decreased TC compared to its base fluid [169,170]. These PCMs are typically in the form of micro-/nano-encapsulated particles or emulsified droplets that undergo solid–liquid phase transitions within the operating temperature range. To produce a PCS, two grades of microencapsulated paraffin wax were dispersed in water by Zhang et al. [58]. Despite the lower TC of the slurry compared with water, DSC measurements confirmed the slurry’s ability to absorb and release latent heat, resulting in increased Effective Heat Capacity (EHC; this parameter considers the cumulative effect of SHC and latent heat of any possible phase transition). Likewise, Han et al. [171] aimed to improve the TC and EHC of poly alpha olefin by indium NPs. The formulated NF consistently exhibited higher TC than the base fluid at all temperatures, and NPs’ ability in phase transition across a range from 110 °C to 157 °C increased the EHC of the base fluid. Yuan et al. [172] synthesized microencapsulated PCMs using a paraffin core and silica–graphene oxide shell, dispersing them in water. These NPs moderately increased EHC before and during the phase transition. Additionally, the insertion of 10 wt.% of paraffin core and silica–graphene oxide shell NPs in water increased TC by 8% at 80 °C. Water NPs were stabilized in perfluoro hexane to enhance their TC by 52%. Under a 20 °C temperature change and due to the phase transition in water, the volumetric EHC of a mixture containing 12% water NPs increased by 126% [29]. Barlak et al. [173] utilized nano-encapsulated nonadecane in water and EG. Although both base fluids showed an increasing TC with increasing NP content, these changes were very small [173]. In another study, Ho et al. [84] used nano-encapsulated eicosane in water and observed a rise in both EHC and TC during the phase transition for an NF containing only 5 mass% of NPs.

### 5.3. In Nanofluids Containing PCM

Simultaneous use of NPs and micro-/nano-encapsulated PCMs can enhance both TC and EHC. A hybrid NF containing Al_2_O_3_ NPs and microencapsulated n-eicosane in water was synthesized to enhance EHC and TC. The addition of MEPCM increased EHC significantly during the phase transition. Despite MEPCM’s negative impact on TC, combining it with Al_2_O_3_ NPs boosted TC by 9.8% compared to water, though viscosity increased notably. Specifically, 18.2% MEPCM raised viscosity by about 60%, and adding 1% more Al_2_O_3_ NPs spiked viscosity by approximately 200% [59]. In similar research, the simultaneous use of 1 mass% MWCNT and 10 mass% microencapsulated paraffin in water showed a maximum of 8% growth in TC [174]. MWCNT NPs were added to a PCS to enhance TC and prevent supercooling. Adding 1% MWCNT to the suspension of 10% nano-encapsulated octadecane PCM improved TC by 3.89% in the solid state and 4.32% in the liquid state of PCM. This enhancement surpasses water’s TC in the liquid PCM state [9]. A 35% PCS with alkyl hydrocarbon was diluted with water, and TiO_2_ and Al_2_O_3_ NPs were added to improve TC [52]. The addition of 0.1% TiO_2_ and Al_2_O_3_ increased TC by 9.4% and 6.3%, respectively; however, TC was still lower than that of water [52].

Despite significant advances in NF-based heat carriers, a balance in thermophysical properties remains elusive. Improvements in either TC or SHC often come at the expense of the other property. While some studies report enhancements in TC and SHC simultaneously, these are frequently accompanied by a substantial increase in viscosity, hindering practical applications.

## 6. Outlooks and Conclusions

A comprehensive review of NFs focusing on SHC, TC, and underlying mechanisms is presented, highlighting key factors that influence their performance. The enhancement of TC is highly dependent on various parameters, such as dispersion quality, NP volume fraction, type, size, shape, choice of base fluid, and operational temperature, all of which play crucial roles. Similarly, SHC enhancement depends on base fluid, NP characteristics, stability, and temperature. However, it is essential to note that improvements in TC often come at the expense of reduced SHC and vice versa. While some studies report simultaneous enhancements in both parameters, these are usually accompanied by an increase in viscosity, posing challenges for practical applications. In heat transfer applications, NFs enhance forced convection, while their performance in natural convection and boiling depends on the interactions between TC, viscosity, and surface roughness. 

Despite advancements, several challenges and opportunities persist in NF research and application:

∘ Stability remains a critical issue, as current surfactants like SDS and SDBS demonstrate limited effectiveness over a wide temperature range, which could impact long-term performance.
∘ NP synthesis methods are often complex and economically unfeasible, highlighting the need for scalable, repeatable, and cost-effective production techniques.
∘ NPs with solid–solid transitions hold the potential to enhance both SHC and TC without significantly increasing viscosity, benefiting thermal energy storage and isothermal applications. This avenue deserves further exploration.
∘ Industrial applications at elevated pressures and temperatures, such as vegetable oil deodorizers, present opportunities for NF utilization to enhance heat recovery in smaller coils. However, current research on thermosyphons is limited to low-temperature and pressure ranges.
∘ While improved thermal properties of NFs can potentially reduce approach temperatures and mitigate issues like hot spots and fouling in heat-sensitive industries, this aspect has been largely overlooked in existing research.

Future research should prioritize strategies that simultaneously enhance both properties while addressing viscosity challenges. Additionally, integrating PCMs with NFs presents a promising pathway for optimizing thermal performance, especially during phase transitions. Continued research and innovation are vital for overcoming existing limitations and unlocking the full potential of NFs in advanced thermal management applications.

## Figures and Tables

**Table 3:** An overview of the conducted research on TC enhancement using various NPs and base fluids.

NP	Base Fluid	%TC Enhancement	%NP Concentration		Ref.
			Volume	Mass	
CNT	Oil	150	1		[47]
SWCNT	Oil	45	1		[71]
CuO	EG	20		4	[52]
CuO	Water	60	5		[73]
Cu	EG	40	0.3		[21]
Cu	Water	74	0.3		[62]
Cu	Water	80	8		[63]
Cu	Transformer oil	45	8		[63]
SiC	Water and EG	15.8–22.9	4–4.2		[15]
Fe_3_O_4_	Water	48	2		[68]
Fe_3_O_4_	Water	24	0.025		[69]
Al_2_O_3_	Water	2–10.8	1		[74]
Al_2_O_3_	Water	9.4–24.3	4		[74]
Al_2_O_3_	Water	30	5		[73]
Ag	Water	35	1.2		[64]
ZnO	EG	14.38		0.048	[75]
ZnO	Water	23.7		0.048	[38]
TiO_2_	Water	20	2		[43]
TiO_2_	Water	15	1.25		[76]
SiO_2_	Water	14.7	0.039		[70]
SiO_2_	Water	31.84	0.02		[31]

**Table 4:** Summary of enhanced forced convection heat transfer in NFs, including the associated increase in flow resistance.

Base Fluid	Micro/ Nanoparticle	Particle%	Reynolds	Heat Transfer Improvement		Induced Flow Resistance		Ref.
				Increased Nu%	Increased Convective Heat Transfer Coefficient %	Increased Pressure Drop %	Increased Friction Factor %	
Water	Al_2_O_3_+MEPCM (1:1)	0.01 vol.%	1500	+13.06% *	-	-	+8.1% *	[150]
Water	MWCNT+MEPCM (1:10)	12.1 mass%	1250		−30% *	+108% *	-	[147]
Water	Nano-encapsulated PCM	3.8 mass%	112	+82%	-	-	+152% *	[84]
Water	MEPCM	10 mass%	1200	-	+10.7% *	60%	-	[151]
Water	MEPCM	2 vol.%	300	+6.8% *	-	173% *	-	[152]
Water	MEPCM	15 vol.%	-	+6.2%	-	+48%	-	[153]
Water	Cu NPs	2 vol.%	16,000	+27.2% *	-	0%	-	[142]
Water	Al_2_O_3_ NPs	2 vol.%	300	-	+11.94%	+35%	-	[37]
Water + EG (40:60)	Graphene nanoplatelets + cellulose nano	0.2 mass%	10,500	+43.8% *	-	+34% *	-	[4]
Water	MWCNT	0.2 mass%	15,000	+35% *	-	23.3%	-	[154]
Water	Ni NPs	0.6 vol.%	15,000	-	+74.5% *	-	+14% *	[155]
Water + EG (60:40)	TiO_2_ NPs	0.02 vol.%	15,000	-	+10.73%	-	+8.73%	[156]

* These numbers are not reported explicitly in the references and have been calculated based on the reported data.

**Table 5:** A summary of the impact of NFs on heat transfer enhancement.

Base Fluid	NP	NP %	% Heat Transfer Improvement	Convection Type	Ref.
Oil	CuO	2 mass%	12.7	Forced	[53]
Water	Al_2_O_3_	1.3 vol.%	19	Forced	[67]
Water	Al_2_O_3_	2 vol.%	11.94	Forced	[37]
Dowtherm	MoSe_2_	0.1 vol.%	11	Forced	[23]
Dowtherm	WSe_2_	0.1 mass%	34	Forced	[124]
Water	PVP/PEG/Cu nanoaggregate	0.01 g/ml	20.86	Forced	[164]
Water	Cu	2 vol.%	60	Forced	[141,142]
Water	Al_2_O_3_	1.25 mass%	40	Boiling	[51]
Perfluoro triethyl amine	Cu	2 vol%	75	Forced	[165]
Water	TiO_2_	5 mass%	35	Natural	[139]
Water	MWCNT	1.1 mass%	22~25	Forced	[147]
Water	Al_2_O_3_-CuO nanocomposite	0.1 vol%	13.56	Forced	[149]

**Table 6:** Summary of the results of simultaneous studies on SHC and TC.

Base Fluid	Micro-/Nanoparticle	% Changes in TC	% Changes in Heat Capacity	Ref.
BaCl_2_ aqueous solution	TiO_2_	+16.74%	−12.4%	[54]
Motor oil	CuO	+6.2%	−23%	[53]
Water	Al_2_O_3_	+11.8% *	−11.05% *	[37]
Water + EG	Graphene	+18%	−8%	[10]
Perfluorohexane	Water	+52%	+126%	[29]
Ionic fluids	MWCNT	+35.54%	+8%	[25]
Water	Hybrid of Al_2_O_3_ and CNT	+20%	+304% *	[34]
DMAc	Graphene	+48%	+18%	[27]
Dowtherm	MoSe_2_	+11%	+7%	[23]
Dowtherm	WSe_2_	+64%	+4.7%	[124]
Molten salt	CNTs	+149.2%	+78.3%	[125]
Molten salt	Al_2_O_3_	+10%	+14.9%	[166]
Silicon	Pd	+8.5%	+5.5%	[28]
Eutectic and azeotropic mixture of biphenyl and diphenyl oxide	Au	+24.6% *	+12% *	[123]
Polyalphaolefin	Graphite	+740% *	+34% *	[167]

* These numbers are not reported explicitly in the references and have been calculated based on the reported data.

## Data Availability

The data presented in this study are available on request from the corresponding author.

## Abbreviations

CNT	Carbon Nanotube	Nu	Nusselt
EG	Ethylene Glycol	PCM	Phase Change Material
EHC	Effective Heat Capacity	PCS	Phase Change Slurry
H&C	Hamilton and Crosser	Pr	Prantl
HTF	Heat Transfer Fluid	Re	Reynolds
MEPCM	Microencapsulated PCM	SHC	Specific Heat Capacity
MWCNT	Multi-Walled CNT	SSA	Specific Surface Area
NF	Nanofluid	TC	Thermal Conductivity
